# Wide – Field optical coherence tomography angiography in florid proliferative diabetic retinopathy

**DOI:** 10.1016/j.ajoc.2023.101976

**Published:** 2023-12-18

**Authors:** Maria Cristina Savastano, Claudia Fossataro, Stanislao Rizzo

**Affiliations:** a Ophthalmology Unit, Fondazione Policlinico Universitario Agostino Gemelli, IRCCS, Rome, Italy; b Ophthalmology Unit, Catholic University of the Sacred Heart, Rome, Italy; c CNR Neuroscience Institute, Pisa, Italy

**Keywords:** Proliferative diabetic retinopathy, Wide - Field optical coherence tomography angiography, Neovascularization

## 1 Case report

**Fig. 1:**
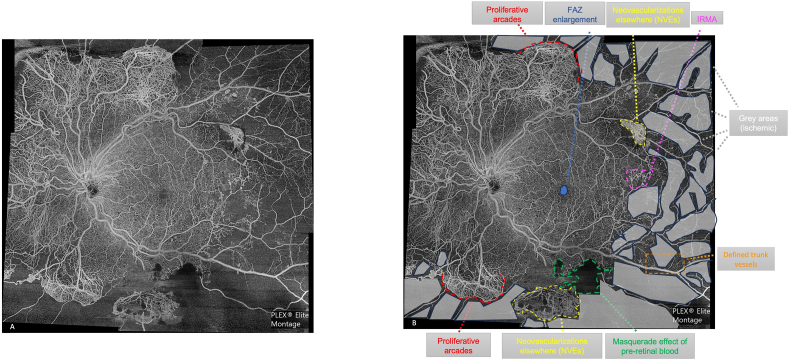
**Wide – field Optical Coherence Tomography Angiography in florid Proliferative Diabetic Retinopathy**: Fig. 1A: wide – field optical coherence tomography angiography showed in detail a case of proliferative diabetic retinopathy (PDR). Fig. 1B highlighted the characteristic PDR features, like proliferative arcades (red), neovascularizations elsewhere (NVEs) (yellow), ischemic areas (grey), foveal avascular zone (FAZ) enlargement (blue). Intraretinal Microvascular Abnormality (IRMA) (pink circle) and defined trunk vessels (orange rectangle) could be observed. Along the inferior retinal vascular arcade, pre-retinal hemorrhage was responsible of masquerade effect (outlined in green). (For interpretation of the references to colour in this figure legend, the reader is referred to the Web version of this article.)

**Fig. 2:**
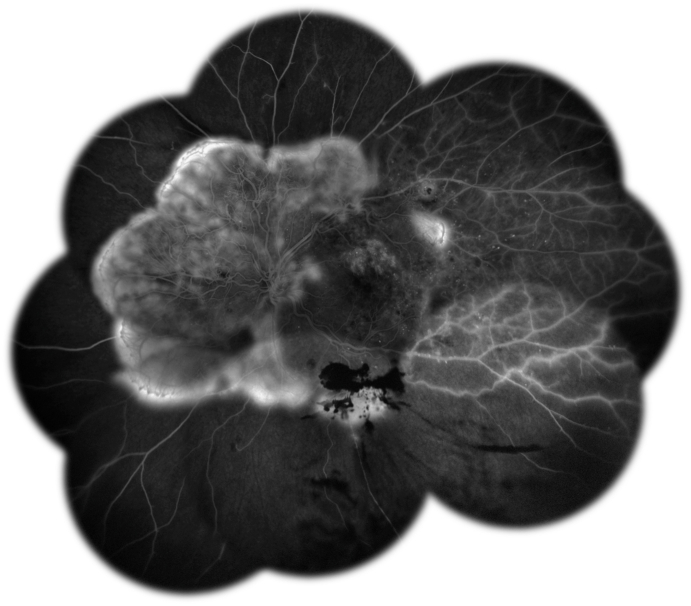
**Montage of Fluorescein Angiography in florid Proliferative Diabetic Retinopathy**: fluorescein angiography of the left eye showed intense hyperfluorescence above the optic disc, consisting in neovascularization on the disc (NVD) and hyperfluorescent neovascular arcades (NVE) in the superonasal, nasal and inferonasal quadrants and superotemporally to the macula. Below the inferotemporal vascular arcade, a mask effect was evident, due to pre-retinal hemorrhage. Several diffuse non perfusion areas were detectable.

A 58-year-old male patient, suffering from type 2 diabetes mellitus, referred our center because of vitreous hemorrhage in the right eye (RE) due to florid proliferative diabetic retinopathy (PDR). The patient underwent a complete ophthalmological examination of the left eye (LE), comprehensive of best correct visual acuity, slit lamp examination, dilated fundus evaluation, retinography, fluorescein angiography (FA), optical coherence tomography (OCT) and wide field OCT – Angiography (OCTA) montage of 5 OCTA volume scans (12 × 12 mm) (PLEX® Elite 9000 2.1; Carl Zeiss Meditec, Dublin, CA, USA). Wide – field OCTA was almost comparable to FA in detecting the main PDR features, such as diffuse non perfusion areas (ischemic) in the mid periphery, associated with severe neovascularizations on the disc (NVD) and elsewhere (NVE) (Fig. 1, Fig. 2). Pre-retinal hemorrhage and neovascular proliferative arcades were evident as well. However, vascular architecture, easily evaluable on OCTA image, was not well-defined on FA, due to dye leakage. Intravitreal injection of anti – VEGF (vascular endothelial growth factor) and photocoagulation laser treatment of the retinal ischemic areas were performed in LE.

## 2 Discussion

Although fluorescein angiography still represents the gold standard to make diagnosis of PDR, the introduction of new wide – field OCTA devices has provided a great support in daily clinical practice, performing even more detailed exams, with no dye injection.^1^ Until a few years ago, only low resolution images, focused on central 3 mm, were available, while nowadays, we can benefit of wide – field high definition scans and of single automated montage of multiple OCTA scans.^2^, ^3^, ^4^

## 3 Conclusion

In our view, in the near future, PDR diagnosis would be easily performed with more advanced wide – field OCT – Angiography devices, which would permit to highlight even the smallest microvascular changes.

## Patient consent

A written consent to publish this case report has been obtained from the patient.

## Financial disclosure

The authors have no financial disclosure to declare.

## Authorship

All authors attest that they meet the current ICMJE criteria for Authorship.

## CRediT authorship contribution statement

**Maria Cristina Savastano:** Conceptualization, Data curation, Formal analysis, Supervision, Writing – original draft, Writing – review & editing. **Claudia Fossataro:** Conceptualization, Data curation, Formal analysis, Investigation, Validation, Writing – original draft, Writing – review & editing. **Stanislao Rizzo:** Conceptualization, Data curation, Investigation, Supervision, Validation, Visualization, Writing – original draft, Writing – review & editing.

## Declaration of competing interest

The authors declare that they have no known competing financial interests or personal relationships that could have appeared to influence the work reported in this paper.
